# The factors affecting the physical development of neonates in pregnant women with or without gestational diabetes mellitus

**DOI:** 10.1371/journal.pone.0251024

**Published:** 2021-04-30

**Authors:** Xiaodi Zhao, Nana Li, Runping Jia, Shumin Chen, Ling Wang

**Affiliations:** 1 College of Public Health, Zhengzhou University, Zhengzhou, Henan, China; 2 Zhengzhou Central Hospital, Zhengzhou, Henan, China; 3 2^nd^ Affiliated Hospital of Zhengzhou University, Henan, China; 4 Faculty of Medicine, Macau University of Science and Technology, Macau, China; University of Insubria, ITALY

## Abstract

**Objectives:**

To explore the factors affecting neonatal physical development in pregnant women with or without gestational diabetes mellitus (GDM).

**Methods:**

The subjects were selected from the pregnant woman giving birth in 2^nd^ Affiliated Hospital of Zhengzhou University, from November 2015 to May 2016. The age, occupation, education level, gestational age, body weight before pregnancy, body weight at delivery, body height, delivery pattern, GDM status of pregnant women and neonatal gender, birth weight (BW), chest circumference (CC), head circumference (HC) and birth length (BL) were collected through medical records and questionnaires. The clinical data were retrospectively analyzed and studied.

**Results:**

The significant differences were found between women with GDM and without GDM in following neonatal variables (*P*<0.05): BW, CC, and HC. GDM status increased the incidence of macrosomia (*OR* = 2.241, 95% *CI*: 1.406–3.573), large CC (*OR* = 2.470, 95% *CI*: 1.687–3.6153). Gestational weight gain (GWG) above IOM guideline was risk factor for macrosomia (*OR* = 1.763, 95% *CI*:1.098–2.833), large HC (OR = 1,584, 95% *CI*: 1.093–2.296) and large CC (*OR* = 1.707, 95% *CI*:1.163–2.506). Underweight was risk factor for short BL (*OR* = 2.543, 95% *CI*:1.161–5.571) and small CC (*OR* = 1.901, 95% *CI*:1.064–3.394). Female neonate was prone to appear short BL(*OR* = 2.831, 95% *CI*: 1.478–5.422) and small HC (*OR* = 2.750, 95% *CI*: 1.413–5.350), and not likely to macrosomia (*OR* = 0.538, 95% *CI*: 0.343–0.843), longer BL (*OR* = 0.584, 95% *CI*: 0.401–0.850), large HC (*OR* = 0.501, 95% *CI*: 0.352–0.713), and (*OR* = 0.640, 95% *CI*: 0.446–0.917). For women with GDM, gestational age was an risk factor of neonatal BW (low BW: *OR* = 0.207, 95% *CI*: 0.085–0.503; macrosomia: *OR* = 1.637, 95% *CI*: 1.177–2.276), BL (short BL: *OR* = 0.376, 95% *CI*: 0.241–0.585; long BL: *OR* = 1.422, 95% *CI*: 1.054–1.919), HC (small HC: *OR* = 0.343, 95% *CI*: 0.202–0.583; large HC: *OR* = 1.399, 95% *CI*: 1.063–1.842) and CC (small CC: *OR* = 0.524, 95% *CI*: 0.374–0.733; large CC: *OR* = 1.485, 95% *CI*: 1.138–1.936).

**Conclusions:**

In our study, gestational age, GDM status, neonatal gender, GWG and pre-pregnancy body mass index (BMI) are associated the abnormal physical development of neonates. In women with GDM, gestational age was correlate with neonatal abnormal physical developments.

## Introduction

Gestational diabetes mellitus (GDM) refers to that the glucose tolerance is abnormal at second or third trimester, but doesn’t include existing diabetes before pregnancy. GDM affects approximately 16.5% of pregnancies worldwide, and the prevalence is rising and correlates with the increase in maternal obesity over recent decades [1].

GDM increases the risk of pregnant complications, such as spontaneous abortion, caesarean delivery, preterm labor, polyhydramnios, urinary tract infection and postoperative/postpartum infection, thromboembolism, and maternal morbidity and mortality postpartum hemorrhage, pregnancy-induced hypertension syndrome, and postpartum type 2 diabetes (T2DM) [2–4]. For the offspring, GDM leads to abnormal fetal development, such as growth retardation, neonatal respiratory distress syndrome, puerperal hypoglycemia, macrosomia, and neonatal asphyxia [5], moreover, it will increase the risk of perinatal death, childhood obesity and adulthood diabetes in the future [6]. Therefore, monitoring and prevention of GDM is very important for pregnant women during pregnancy. The World Health Organization (WHO) established that GDM should be diagnosed by a 75 g OGTT test [7]. Some studies have found that GDM was associated with birth number, age, family history of diabetes, previous GDM, overweight, obesity [8]. In addition, pre-pregnancy body mass index (BMI) and gestational weight gain (GWG) are associated with maternal nutrition.

Neonatal birth weight (BW), birth length (BL), head circumference (HC), and chest circumference (CC) are the common indicators of newborn physical development. Abnormal physical development increases the risk of respiratory disease and mental retardation in infancy, and is even associated with hypertension and diabetes in the future for the offspring [9, 10]. Low BW increases neonatal mortality, while macrosomia may increase the incidence of dystocia, birth injury, postpartum hemorrhage, and obesity in adolescence and adulthood [11]. BL is mainly affected by heredity and intrauterine growth and development level [12, 13]. CC is often related to the development of baby’s chest, lung and subcutaneous fat. Neonatal HC is often closely related to brain development. The physical development index of newborn can reflect the growth status of newborn. The physical development of newborns is closely related to pregnant women. In this study, we investigated the factors affecting the physical development of neonates among pregnant women with or without GDM.

## Methods

### Subjects

This study was conducted in the 2^nd^ Affiliated Hospital of Zhengzhou University from November 2015 to May 2016. Women about to give birth eligible for the study enrolment: 20–40 years old, singleton pregnancy, and partus maturus. Women have been diagnosed with diabetes, hyperthyroidism, hypertension, and heart disease, refusal to participate in the study, and incomplete medical records were excluded. Fetal congenital malformations and premature delivery were excluded.

This research was consistent with the Helsinki Declaration, and Zhengzhou University Life Science Ethics Review Board granted clearance for the study (ZZUIRB 2021–07).

### Information collection

The basic information obtained included: (1) maternal characteristics including age, education level, occupation, body height, pre-pregnant body weight, body weight at delivery, gestational age, delivery pattern, and GDM status; (2) newborn characteristics including gender, BW, BL, HC and CC. The basic information of the subjects was collected through medical record and questionnaires and informed consents were obtained.

Maternal self-reported pre-pregnant weight and height were used to calculate the pre-pregnancy BMI [calculated as weight (kg)/height (m)^2^]. According to the BMI judgment criteria for adults from World Health Organization [14], the BMI were divided into: underweight (<18.5 kg/m^2^), normal weight (18.5 kg/m^2^≤BMI<25.0 kg/m^2^), overweight (25.0 kg/m^2^≤BMI<30.0 kg/m^2^), and obesity (BMI≥30.0 kg/m^2^). In this study, because the case number of overweight and obesity was relatively small, the two categories were combined as overweight/obesity.

GWG was calculated by subtracting each woman’s pre-pregnant weight from her weight at delivery. The recommended GWG from Institute of Medicine (IOM) (2009) is to gain 12.5–18 kg, 11.5–16 kg, 7–11.5 kg, and 5–9 kg for underweight, normal weight, overweight, and obesity women, respectively. GWG was divided into the following categories: (1) below; (2) within; and (3) above IOM guidelines by referring the IOM recommendations (2009).

The classification of neonatal physical development based on BW, BL, HC, CC were as follows: (1) < 2500 g, low BW; 2500 g ≤ BW < 4000 g, normal BW; ≥ 4000 g, macrosomia; (2) < 10^th^ percentile, short BL; 10^th^-90^th^ percentile, normal BL; >90^th^ percentile, long BL; (3) < 10^th^ percentile, small HC;10^th^-90^th^ percentile, normal HC; > 90^th^ percentile, large HC; (4) < 10^th^ percentile, small CC: 10^th^-90^th^ percentile, normal CC; > 90^th^ percentile, large CC.

GDM was assessed using oral glucose tolerance test (OGTT). All pregnant women underwent a 2-h 75-g oral glucose tolerance test (OGTT) at 24–28 weeks of pregnancy.

The diagnostic criteria for GDM in this study were based on the World Health Organization (WHO) “Diagnostic Criteria and classification of Hyperglycemia first detected in pregnancy” [7]: fasting plasma glucose 5.1–6.9 mmol/l (92–125 mg/dl); 1-hour plasma glucose ≥ 10.0 mmol/l (180 mg/dl) following a 75 g oral glucose load; 2-hour plasma glucose ≥ 8.5–11.0 mmol/l (153–199 mg/dl) following a 75 g oral glucose load. The plasma glucose level met any one of the three would be considered as GDM.

### Statistical analysis

EpiData 3.1 was used for data entry. Continuous variables were presented as the mean±SD, and categorical variables were calculated as the frequencies and percentages. Differences between continuous variables were determined using t-test. The chi-squared test was used to analyze differences between categorical variables. The chi-squared test was also used for analyzing the influence of neonatal gender, GDM status, education level, occupation, classification of pre-pregnant BMI, and GWG classes on neonatal BW, BL, HC, CC, and PI. One-way analysis of variance (ANOVA) was used for analyzing the influence of maternal age and gestational age on neonatal BW, BL, HC, CC, and PI. And logistic regression was used for multivariate analyses based on the factors associated with neonatal BW, BL, HC, CC.

## Results

### General information

Of the 1108 women enrolled in the study, 308 women were with GDM and 800 women were not. The characteristics of the mothers and newborns are presented in Table 1. Women with GDM were older, and the mean of pre-pregnancy BMI (22.96 ± 3.40 vs 21.40 ± 2.85) and cesarean section rate were higher than women without GDM (*P* <0.05), nevertheless, the mean of GWG (14.63 ± 5.22 vs 16.17 ± 4.94) was lower in women with GDM than that women without GDM (*P* <0.05). Furthermore, the proportion of overweight/obesity (23.7% vs 10.1%) in women with GDM was higher than that in women without GDM (*P* <0.05), and the proportion of underweight (7.1% vs 13.1%) and normal weight (69.2% vs 76.8%) were lower than that in women without GDM (*P* <0.05). The proportion of GWG below IOM guideline (20.1% vs 13.9%) was lower in women with GDM than that in women without GDM (*P* <0.05). The neonatal BW (3.53 ± 0.45 vs 3.38 ± 0.39), CC (34.13 ± 1.73 vs 33.63 ± 1.63), and HC (34.23±1.45 vs 33.93±1.50) were significantly larger in newborns GDM-exposed than that unexposed neonates (*P* <0.05). There was no difference in neonatal gender between women with GDM and women without GDM.

**Table 1:** Basic information of pregnant women and newborns.

Variables	Women with GDM (308)	Women without GDM (800)	*t*/*χ*^2^	^*P*^
**Maternal** **parameters**				
Maternal age (years)	31.69±4.43*	29.89±4.22	-6.145	<0.001
Pre-pregnancy BMI (kg/m^2^)	22.96±3.40*	21.40±2.85	-7.136	<0.001
GWG (kg)	14.63±5.22*	16.17±4.94	4.550	<0.001
Gestational age (weeks)	39.22±1.05	39.31±1.05	1.304	0.193
Pre-pregnancy BMI class				
underweight	22(7.1%)*	105(13.1%)	38.152	<0.001
Normal weight	213(69.2%)*	614(76.8%)		
Overweight/obesity	73(23.7%)*	81(10.1%)		
GWG class				
Below IOM guideline	62(20.1%)*	111(13.9%)	6.968	0.031
Within IOM guideline	122(39.6%)	326(40.8%)		
Above IOM guideline	124(40.3%)	363(45.4%)		
Occupation				
Medical and health	26(8.4%)	61(7.6%)	8.545	0.074
Education	29(9.4%)	74(9.3%)		
Business	32(10.4%)	80(10.0%)		
Administrative	141(45.8)	307(38.4)		
Others	80(26.0%)	278(34.8%)		
Maternal education				
Middle school or below	32(10.4%)	107(13.4%)	3.018	0.221
High school or junior college	94(30.5%)	262(32.8%)		
Bachelor or above	182(59.1%)	431(53.9%)		
Delivery pattern				
Vaginal delivery	105(34.1%)	328(41.0%)	4.459	0.035
Cesarean	203(65.9%)*	472(59.0%)		
**Neonatal** **parameters**				
Males, n (%)	51.90%	54.10%	0.424	0.515
BW (kg)	3.53±0.45*	3.38±0.39	-5.191	<0.001
BL (cm)	50.69±1.80	50.47±1.78	-1.921	0.055
CC (cm)	34.13±1.73*	33.63±1.63	-4.506	<0.001
HC (cm)	34.23±1.45*	33.93±1.50	-3.029	0.003

Note: GDM: gestational diabetes mellitus; GWG: gestational weight gain; BMI: body mass index. IOM: Institute of Medicine; BW: birth weight; BL: birth length; CC: chest circumference; HC: head circumference.

*Compared with women without GDM, *P*<0.05.

There was obvious difference in the physical development between male and female infants in Table 2. When stratified by sex, we found the significant differences in BW, BL, HC and CC between male and female neonates. The BW, BL, HC and CC were higher in male neonates compared with females (*P* <0.05).

**Table 2:** Birth outcomes of male and female neonates.

	Male (mean±SD)	Female (mean±SD)	*t*	*P*
BW(g)	3464.26±425.76	3359.54±431.42	4.035	<0.001
BL(cm)	50.66±2.72	50.21±1.86	4.834	<0.001
CC(cm)	33.84±1.76	33.59±1.69	2.323	0.02
HC(cm)	34.20±1.53	33.74±1.53	4.969	<0.001

Note: BW: birth weight; BL: birth length; CC: chest circumference; HC: head circumference.

### Factors associated with neonatal BW

In whole subjects, low BW (1.3%), normal weight (90%) and macrosomia (8.7%). In women without GDM, low BW (1%), normal weight (92%) and macrosomia (7%). In women with GDM, low BW (1.9%), normal weight (84.7%) and macrosomia (13.3%). Factors influencing the BW of newborns was shown in Table 3. GDM status, pre-pregnancy BMI class, GWG class, and gestational age were associated with BW in whole subjects (*P* <0.05). In women without GDM, gestational age was associated with BW (*P* <0.05). In women with GDM, GWG class and gestational age were associated with BW (*P* <0.05). A multivariate analysis was performed to further investigate whether these factors had an impact on neonatal BW.

**Table 3:** Parameters associated with neonatal BW in the whole subjects, and in women without/with GDM.

	Whole subjects		Women without GDM		Women with GDM	
	*χ*^2^/*F*	*P*	*χ*^2^/*F*	*P*	*χ*^2^/*F*	*P*
Neonatal gender	4.297	0.117	5.202	0.074	0.497	0.780
GDM status	13.106	<0.001	-	-	-	-
Education level	2.613	0.856	1.223	0.874	8.872	0.064
Occupation	14.846	0.138	7.081	0.528	4.958	0.762
Pre-pregnancy BMI class	23.549	<0.001	9.843	0.131	4.008	0.405
GWG class	16.596	<0.001	8.606	0.072	16.419	0.003
Maternal age (years)	1.111	0.319	1.131	0.312	0.797	0.740
Gestational age (weeks)	6.078	<0.001	2.718	0.013	5.760	<0.001

Note: GDM: gestational diabetes mellitus; GWG: gestational weight gain; BMI: body mass index; BW: birth weight.

**Fig 1:**
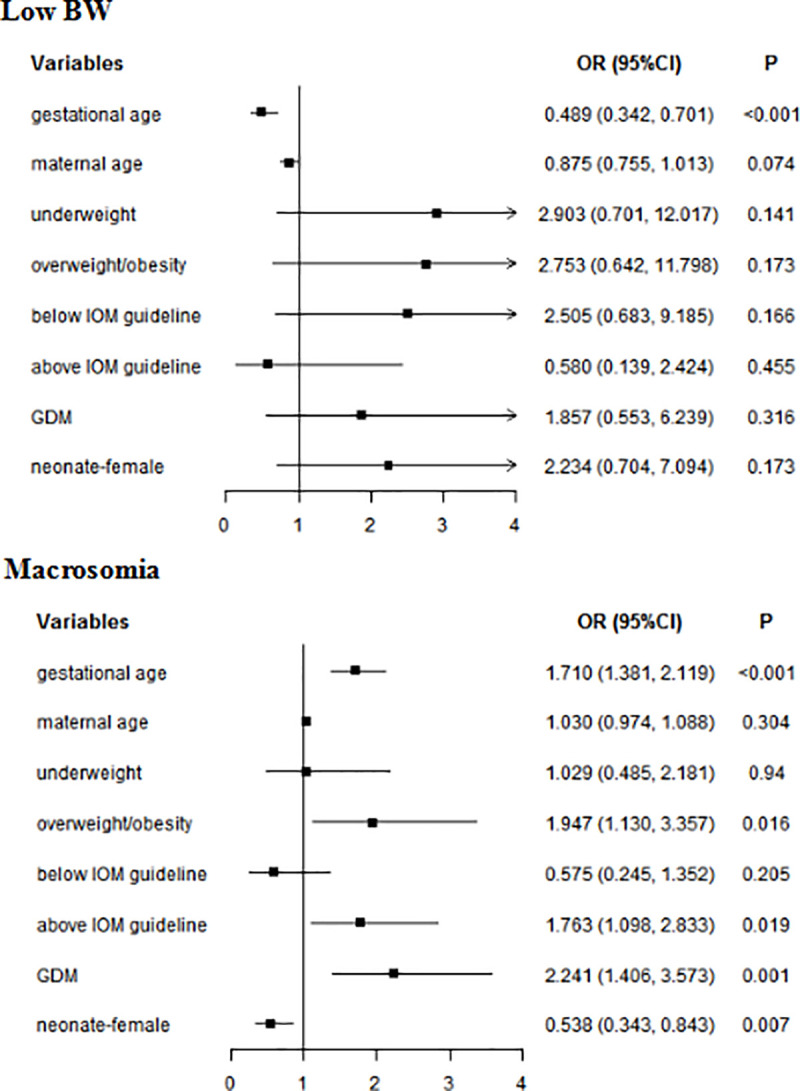
Multivariate analyses of neonatal BW in whole subjects. GDM: gestational diabetes mellitus; BW: birth weight; Normal weight was used as a reference for underweight and overweight/obesity; Within IOM guideline was used as a reference for Below IOM guideline and Above IOM guideline.

The above parameters and the factors that might affect BW were took into account in the Multivariate analyses. In whole subjects, gestational age was associated with BW (low BW: *OR* = 0.489, 95% *CI*: 0.342–0.701; macrosomia: OR = 1.710, 95% *CI*: 1.381–2.119). Overweight/obesity (*OR* = 1.947, 95% *CI*: 1.130–3.357), GWG above IOM guideline (*OR* = 1.763, 95% *CI*:1.098–2.833), and GDM (*OR* = 2.241, 95% *CI*: 1.406–3.573) were risk factors for a macrosomia. Female neonate was not more likely to appear a macrosomia (*OR* = 0.538, 95% *CI*: 0.343–0.843) (Fig 1). In the women without GDM, gestational age (*OR* = 1.673, 95% *CI*: 1.279–2.189) and GWG above IOM guideline (*OR* = 1.919, 95% *CI*: 1.046–3.519) increased the risk of macrosomia. Female was not prone to macrosomia (*OR* = 0.483, 95% *CI*: 0.268–0.870). In women with GDM, gestational age (low BW: *OR* = 0.207, 95% *CI*: 0.085–0.503; macrosomia: *OR* = 1.637, 95% *CI*: 1.177–2.276) was associated with BW (Table 4).

**Table 4:** Multivariate analyses of neonatal BW in women without/with GDM.

	Low BW		Macrosomia	
	*OR*	*P*	*OR*	*P*
**Women with GDM**				
Gestational age	0.207 (0.085–0.503)	0.001	1.637 (1.177–2.276)	0.003
neonatal gender				
male	REF		REF	
female	2.696 (0.332–21.87)	0.353	0.707 (0.356–1.403)	0.321
GWG classes				
Within IOM guideline	REF		REF	
Below IOM guideline	4.025 (0.483–33.565)	0.198	0.379 (0.105–1.372)	0.139
Above IOM guideline	2.230E-9 (2.230E-9-2.230E-9)	-	1.452 (0.708–2.977)	0.308
**Women without GDM**				
Gestational age	0.763 (0.459–1.267)	0.296	1.673 (1.279–2.189)	<0.001
neonatal gender				
male	REF		REF	
female	2.019 (0.470–8.675)	0.345	0.483 (0.268–0.870)	0.015
GWG classes				
Within IOM guideline	REF		REF	
Below IOM guideline	1.506E-8 (1.506E-8-1.506E-8)	-	2.692E-8 (0-+∞)	0.998
Above IOM guideline	1.178 (0.278–4.995)	0.824	1.919 (1.046–3.519)	0.035

Note: GDM: gestational diabetes mellitus; GWG: gestational weight gain; IOM: Institute of Medicine; BW: birth weight.

REF: Reference group.

### Factors associated with neonatal BL

The factors influencing neonatal BL are shown in Table 5. Neonatal gender, GWG class, and gestational age were associated with neonatal BL in whole subjects (*P* <0.05). Neonatal gender, GWG class, maternal age and gestational age were associated with BL in women without GDM (*P* <0.05). Only Gestational age was related to BL in women without GDM (*P* <0.05).

**Table 5:** Parameters associated with neonatal BL in the total population, and in women without/with GDM.

	Whole subjects		Women without GDM		Women with GDM	
	*χ*^2^/*F*	*P*	*χ*^2^/*F*	*P*	*χ*^2^/*F*	*P*
Neonatal gender	12.349	0.002	8.303	0.016	5.149	0.076
GDM status	1.299	0.522	-	-	-	-
Education level	10.443	0.537	9.259	0.055	8.728	0.068
Occupation	9.067	0.526	8.807	0.359	4.955	0.758
Pre-pregnancy BMI class	7.304	0.294	10.878	0.092	1.514	0.824
GWG class	11.685	0.020	13.548	0.009	6.227	0.183
Maternal age	1.328	0.126	1.878	0.015	0.857	0.661
Gestational age	9.734	<0.001	8.663	<0.001	6.621	<0.001

Note: GDM: gestational diabetes mellitus; GWG: gestational weight gain; BMI: body mass index; BL: body length.

*P*<0.05 indicates a significant difference.

**Fig 2:**
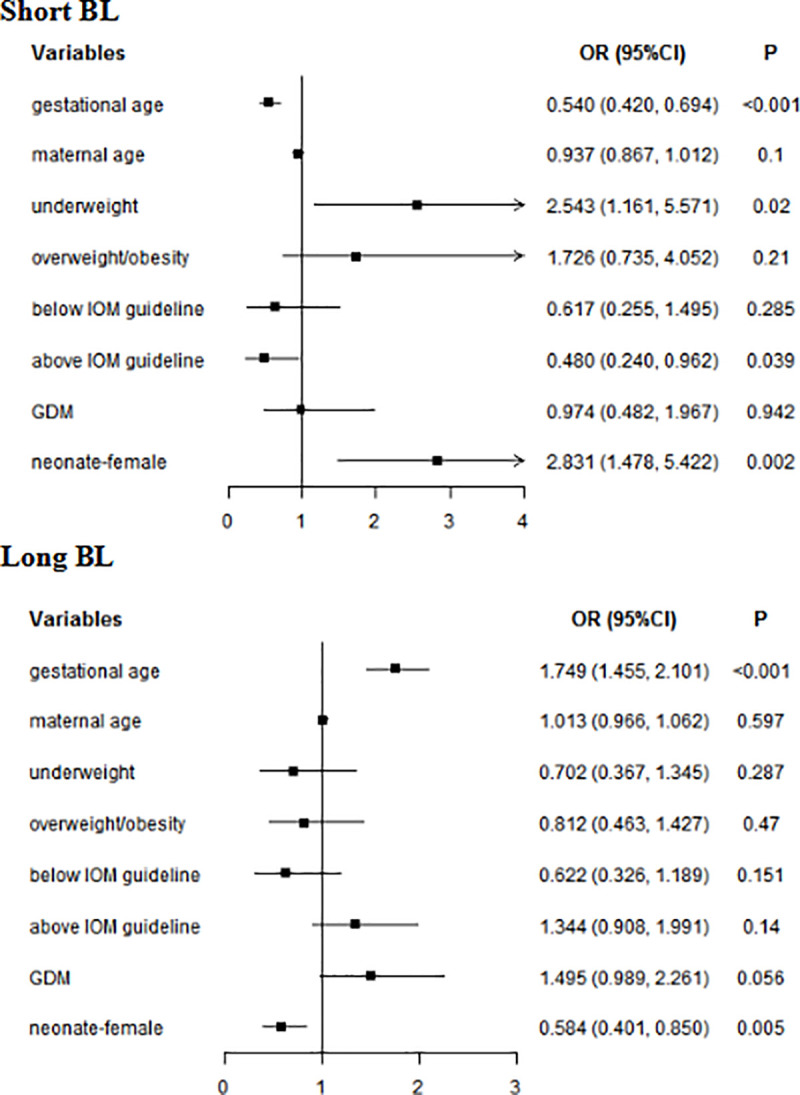
Multivariate analyses of neonatal BL in whole subjects. GDM: gestational diabetes mellitus; BL: birth length; Normal weight was used as a reference for underweight and overweight/obesity; Within IOM guideline was used as a reference for Below IOM guideline and Above IOM guideline.

In multivariate analyses, for whole subjects, gestational age (short BL: *OR* = 0.540, 95% *CI*: 0.420–0.694; long BL: *OR* = 1.749, 95% *CI*: 1.455–2.101) and female (short BL: *OR* = 2.831, 95% *CI*: 1.478–5.422; long BL: *OR* = 0.584, 95% *CI*: 0.401–0.850) were associated with BL. Underweight increased the risk of short BL (*OR* = 2.543, 95% *CI*: 1.161–5.571), while GWG above IOM guideline reduced the risk of short BL (*OR* = 0.480, 95% *CI*: 0.240–0.962) (Fig 2). In women without GDM, gestational age (short BL: *OR* = 0.630, 95% *CI*: 0.473–0.839; long BL: *OR* = 1.974, 95% *CI*: 1.582–2.463) and female (short BL: *OR* = 2.815, 95% *CI*: 1.291–6.135); long BL: *OR* = 0.592, 95% *CI*: 0.375–0.933) were associated with BL. Underweight was a risk factor for short BL(*OR* = 3.510, 95% *CI*: 1.464–8.414). In women with GDM, gestational age was associated with BL (short BL: *OR* = 0.376, 95% *CI*: 0.241–0.585; long BL: *OR* = 1.422, 95% *CI*: 1.054–1.919). Female neonate (*OR* = 3.892, 95% *CI*: 1.060–14.289) was more likely to short HL (Table 6).

**Table 6:** Multivariate analyses of neonatal BL in women with/without GDM.

	Short BL		Long BL	
	*OR*	*P*	*OR*	*P*
**Women with GDM**				
Gestational age	0.376 (0.241–0.585)	<0.001	1.422 (1.054–1.919)	0.021
neonatal gender				
male	REF		REF	
female	3.892 (1.060–14.289)	0.041	0.570 (0.294–1.102)	0.095
**Women without GDM**				
Gestational age	0.630 (0.473–0.839)	0.002	1.974 (1.582–2.463)	<0.001
neonatal gender				
male	REF		REF	
female	2.815 (1.291–6.135)	0.009	0.592 (0.375–0.933)	0.024
GWG classes				
Within IOM guideline	REF		REF	
Below IOM guideline	0.285 (0.034–2.406)	0.249	0.563 (0.068–4.633)	0.593
Above IOM guideline	0.494 (0.232–1.052)	0.067	1.593 (0.995–2.550)	0.053
pre-pregnant BMI				
normal weight	REF		REF	
underweight	3.510 (1.464–8.414)	0.005	0.811 (0.376–1.751)	0.594
overweight/obesity	1.910 (0.613–5.954)	0.264	1.251 (0.578–2.707)	0.569

Note: GDM: gestational diabetes mellitus; GWG: gestational weight gain; BMI: body mass index. IOM: Institute of Medicine; BL: body length.

REF: reference group.

### Factors associated with neonatal HC

Maternal pre-pregnancy BMI class, GWG class, gestational age, and neonatal gender were associated with neonatal HC in whole subjects and women without GDM (P < 0.05). In women with GDM, GWG class and gestational age was associated with neonatal HC (Table 7).

**Table 7:** Parameters associated with neonatal HC in the total population, and in women without/with GDM.

	Whole subjects		Women without GDM		Women with GDM	
	*χ*^2^/*F*	*P*	*χ*^2^/*F*	*P*	*χ*^2^/*F*	*P*
Neonatal gender	17.041	<0.001	17.343	<0.001	2.862	0.239
GDM status	2.778	0.249	-	-	-	-
Education level	4.721	0.580	3.591	0.464	6.830	0.145
Occupation	6.267	0.792	9.198	0.326	5.301	0.725
Pre-pregnancy BMI class	21.460	0.002	15.358	0.018	4.238	0.375
GWG class	10.824	0.029	10.451	0.033	11.944	0.018
Maternal age	0.975	0.501	0.914	0.569	0.847	0.675
Gestational age	7.336	<0.001	6.921	<0.001	5.147	<0.001

Note: GDM: gestational diabetes mellitus; GWG: gestational weight gain; BMI: body mass index; HC: head circumference.

*P*<0.05 indicates a statistically significant difference.

**Fig 3:**
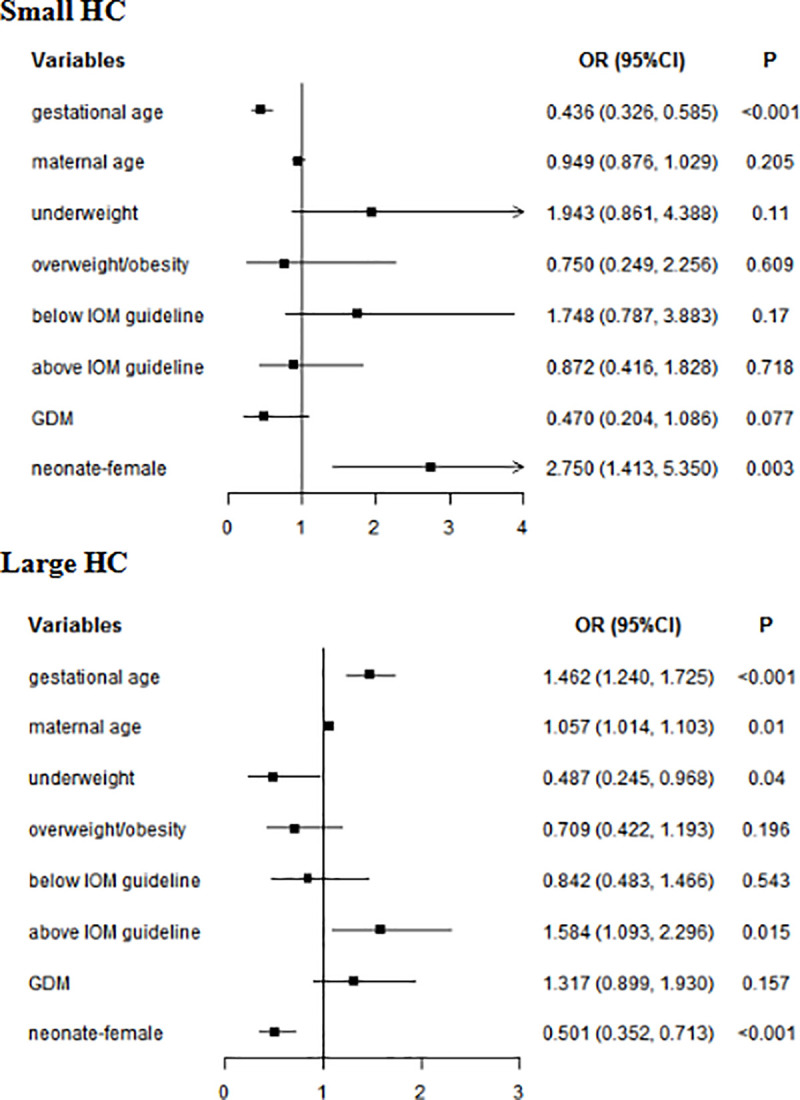
Multivariate analyses of neonatal HC in whole subjects. GDM: gestational diabetes mellitus; HC: head circumference; Normal weight was used as a reference for underweight and overweight/obesity; Within IOM guideline was used as a reference for Below IOM guideline and Above IOM guideline.

In multivariate analyses, for whole subjects, gestational age (small HC: *OR* = 0.499, 95% CI: 0.358–0.696; large HC: *OR* = 1.522, 95% *CI*: 1.242–1.867) and female neonate (small HC: *OR* = 1.057, 95% *CI*: 1.014–1.103; large HC: *OR* = 0.501, 95% *CI*: 0.352–0.713) were related to neonatal HC. Maternal age (*OR* = 1.075, 95% *CI*: 1.019–1.133) increased the risk of neonatal large HC. Underweight reduced the risk of large neonatal HC (*OR* = 0.487, 95% *CI*: 0.245–0.968), while GWG above IOM guideline was a risk factor of large neonatal HC (*OR* = 1.584, 95% *CI*: 1.093–2.296) (Fig 3). In women without GDM, gestational age (small HC: *OR* = 0.499, 95% *CI*: 0.358–0.696; large HC: *OR* = 1.522, 95% *CI*: 1.242–1.867) and Female neonate (small HC: *OR* = 3.621, 95% *CI*: 1.670–7.850; large HC: *OR* = 0.466, 95% *CI*: 0.303–0.716) were related to neonatal HC. GWG above IOM guideline (*OR* = 1.813, 95% *CI*: 1.173–2.801) increased the risk of large HC. In women with GDM, gestational age (small HC: *OR* = 0.343, 95% *CI*: 0.202–0.583; large HC: *OR* = 1.399, 95% *CI*: 1.063–1.842) was associated with neonatal HC (Table 8).

**Table 8:** Multivariate analyses of neonatal HC in women without/with GDM.

	Small HC		Large HC	
	*OR*	*P*	*OR*	*P*
**Women with GDM**				
Gestational age	0.343 (0.202–0.583)	<0.001	1.399 (1.063–1.842)	0.017
neonatal gender				
male	REF		REF	
female	0.987 (0.233–4.173)	0.986	0.571 (0.310–1.049)	0.071
**Women without GDM**				
Gestational age	0.499 (0.358–0.696)	<0.001	1.522 (1.242–1.867)	<0.001
age	0.907 (0.828–0.994)	0.036	1.075 (1.019–1.133)	0.008
neonatal gender				
male	REF		REF	
female	3.621 (1.670–7.850)	0.001	0.466 (0.303–0.716)	0.001
GWG classes				
Within IOM guideline	REF		REF	
Below IOM guideline	2.154 (0.542–8.563)	0.276	0.403 (0.052–3.136)	0.385
Above IOM guideline	0.789 (0.380–1.638)	0.525	1.813 (1.173–2.801)	0.007

Note: GDM: gestational diabetes mellitus; GWG: gestational weight gain; IOM: Institute of Medicine; HC: head circumference.

REF: reference group.

### Factors associated with neonatal CC

GDM status, pre-pregnancy BMI class and GWG class and gestational age were associated with CC in whole subjects (*P* <0.05). In women without GDM, pre-pregnancy BMI class and GWG class and gestational age were associated with CC (*P* <0.05). In women with GDM, GWG class and gestational age were associated with neonatal CC (*P* <0.05) (Table 9).

**Table 9:** Parameters associated with neonatal CC in the total population, and in women without/with GDM.

	Whole subjects		Women without GDM		Women with GDM	
	*χ*^2^/*F*	*P*	*χ*^2^/*F*	*P*	*χ*^2^/*F*	*P*
Neonatal gender	3.168	0.205	3.740	0.154	1.658	0.436
GDM status	20.912	<0.001	-	-	-	-
Education level	7.807	0.253	7.851	0.097	3.901	0.420
Occupation	14.350	0.158	13.272	0.103	12.723	0.122
Pre-pregnancy BMI class	25.916	<0.001	28.000	<0.001	4.555	0.336
GWG class	26.940	<0.001	20.662	<0.001	17.623	0.001
Maternal age	1.069	0.371	1.266	0.194	0.818	0.713
Gestational age	10.050	<0.001	9.075	<0.001	6.562	<0.001

Note: GDM: gestational diabetes mellitus; GWG: gestational weight gain; BMI: body mass index; CC: chest circumference.

*P*<0.05 indicates a statistically significant difference.

**Fig 4:**
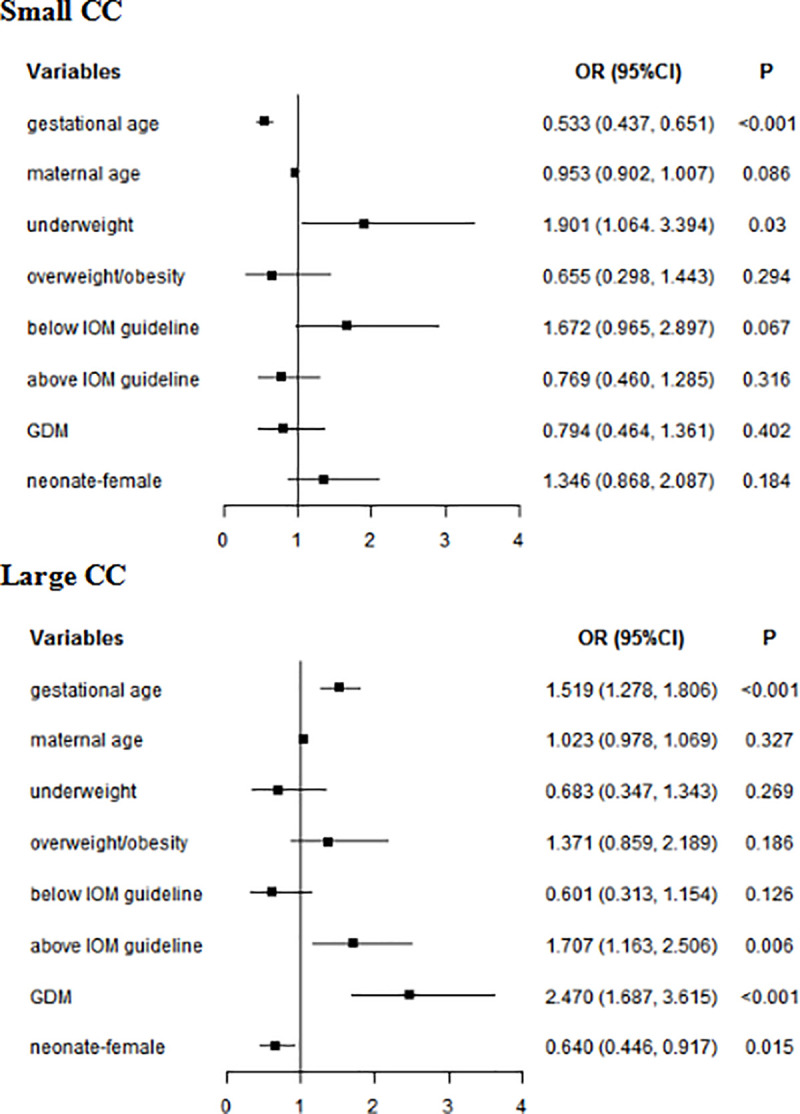
Multivariate analyses of neonatal CC in whole subjects. GDM: gestational diabetes mellitus; CC: chest circumference; Normal weight was used as a reference for underweight and overweight/obesity; Within IOM guideline was used as a reference for Below IOM guideline and Above IOM guideline.

In multivariate analyses, for whole subjects, gestational age (small CC: *OR* = 0.533, 95% *CI*: 0.437–0.651; large CC: *OR* = 1.519, 95% *CI*: 1.278–1.806) was associated with neonatal CC. Underweight increased the risk of small CC (*OR* = 1.901, 95% *CI*: 1.064–3.394). GWG above IOM guideline (*OR* = 1.707, 95% *CI*: 1.163–2.506) and GDM (*OR* = 2.470, 95% *CI*: 1.687–3.615) and GDM (*OR* = 2.470, 95% *CI*: 1.687–3.615) increased the risk of large CC. Female (*OR* = 0.640, 95% *CI*: 0.446–0.917) reduced the risk of large CC (Fig 4). In women without GDM, gestational age (small CC: *OR* = 0.559, 95% *CI*: 0.439–0.712; large CC: *OR* = 1.514, 95% *CI*: 1.216–1.886) was associated with neonatal CC. Underweight increased the risk of small CC (*OR* = 2.619, 95% *CI*: 1.398–4.907). GWG above IOM guideline was a risk factor for large CC (*OR* = 1.738, 95% *CI*: 1.061–2.845). In women with GDM, gestational age (small CC: *OR* = 0.524, 95% *CI*: 0.374–0.733; large CC: *OR* = 1.485, 95% *CI*: 1.138–1.936) was associated with neonatal CC. GWG below IOM guideline (*OR* = 3.644, 95% CI: 1.223–10.855) was a risk factor of small CC (Table 10).

**Table 10:** Multivariate analyses of neonatal CC in women without/with GDM.

	Small CC		Large CC	
	*OR*	*P*	*OR*	*P*
**Women with GDM**				
Gestational age	0.524 (0.374–0.733)	<0.001	1.485 (1.138–1.936)	0.004
Neonatal gender				
male	REF		REF	
female	0.861 (0.344–2.151)	0.748	0.694 (0.395–1.220)	0.204
GWG classes				
within IOM guideline	REF		REF	
below IOM guideline	3.644 (1.223–10.855)	0.020	0.300 (0.629–0.262)	1.512
above IOM guideline	1.314 (0.392–4.407)	0.658	0.348 (1.366–0.730)	2.447
**Women without GDM**				
Gestational age	0.559 (0.439–0.712)	<0.001	1.514 (1.216–1.886)	<0.001
Neonatal gender				
male	REF		REF	
female	1.575 (0.952–2.606)	0.077	0.656 (0.413–1.044)	0.076
GWG class				
within IOM guideline	REF		REF	
below IOM guideline	0.733 (0.214–2.505)	0.620	0.853 (0.102–7.112)	0.883
above IOM guideline	0.806 (0.482–1.346)	0.409	1.738 (1.061–2.845)	0.028
Pre-pregnancy BMI class				
normal weight	REF		REF	
underweight	2.619 (1.398–4.907)	0.003	0.384 (0.132–1.112)	0.078
overweight/obesity	0.619 (0.212–1.812)	0.382	1.905 (0.970–3.741)	0.061

Note: GDM: gestational diabetes mellitus; GWG: gestational weight gain; IOM: Institute of Medicine; CC: chest circumference.

REF: reference group.

## Discussion

Our study found that gestational age, GDM status, neonatal gender, GWG, and pre-pregnancy BMI were associated with neonatal physical development. In whole subjects, gestational age was associated with neonatal BW, BL,HC and CC. Male neonate had a higher risk of macrosomia, long BL, large HC and large CC. Underweight increased the risk of short BL and small CC. GWG above IOM guideline and GDM increased the risk of macrosomia and large CC, besides excessive GWG increased the risk of large HC. In women without GDM, gestational age was also observed to correlate with neonatal BW, BL, HC and CC. Male neonate increased the risk of macrosomia, long BL, large HC. GWG above IOM guideline increased the risk of macrosomia, large CC, and large HC. Underweight increased the risk of short BL and small CC. However, in women with GDM, gestational age was correlate with neonatal BW, BL,HC and CC. Female increased the risk of short BL, and underweight increased the risk of small CC.

The neonatal BW, CC, and HC were higher in women with GDM than that in women without GDM. Multivariate analyses of our study showed that GDM increased the risk of macrosomia and large CC. Studies have found that a macrosomia is a 3-fold higher rate in pregnant women with GDM compared to women without GDM [15]. A survey in 2011 showed that the incidence of macrosomia was 7.3% in China [16]. In present study, the proportion of macrosomia in the whole subjects was 8.8%, which is higher than the national average. However, the incidence of macrosomia in women with GDM was 13.30%, which was significantly higher than that in women without GDM, and it might be associated with fetal weight gain due to maternal hyperglycemia during pregnancy. This is associated with insulin resistance, in the early pregnancy, insulin sensitivity increases, which promotes the growth of adipose and energy storage. With the progress of pregnancy, the surge of hormones including estrogen, progesterone, leptin, cortisol and placenta prolactin might further enhance insulin resistance. On the one hand, pancreas islet cells become hypertrophy and hyperplasia, on the other hand, glucose stimulates insulin secretion to adapt these changes, resulting in elevated blood glucose, even developing gestational diabetes. For the fetus, increased blood sugar enters the fetal circulation through the placenta, then stores as body fat in the fetus, finally leading to giant babies [1, 15, 17]. Macrosomic fetuses will appear subcutaneous fat deposits in the abdomen and interscapular areas in women with GDM [18], which maybe lead to the occurrence of large CC.

In our study, the proportion of overweight/obesity was higher, but GWG was lower in pregnant women with GDM than that in pregnant women without GDM. Overweight/obese is a risk factor of GDM [19, 20]. Pre-pregnancy BMI and GWG influence fetal growth [21]. A woman with GDM requires dietetic counseling for medical nutrition therapy, which is of paramount importance to achieve glycemic control [22]. The present study presents dietary modifications, exercise and glucose monitoring are the first line of treatment for GDM [23]. More than 90 percent of the pregnant women in our study controlled their blood sugar through diet and exercise, and very few patients with poor blood sugar control received insulin therapy. Exercise is associated with a lower BMI, as well as lower GWG. Bruno et al [24] showed that the adherence to a personalized, hypocaloric, low-glycemic, low-saturated fat diet started early in pregnancy could prevent GDM occurrence in women with BMI ≥ 25 kg/m^2^. Sorbye et al. [25] showed that lowering pre-pregnancy BMI could reduce the risk of a recurrence of GDM in overweight/obese women (BMI ≥25). In addition, some non-drug effects on GDM are also of concern, and such as myo-inositol supplementation and vitamin D maybe reduce the risk of GDM [22, 26].

Multivariate analyses results showed that excessive GWG and underweight were associated with neonatal abnormal physical developments in women without GDM. Pre-pregnancy BMI reflects nutritional status before pregnancy. Pre-pregnant underweight, overweight and/or obesity can lead to adverse pregnancy outcomes [27]. Other studies have found that pre-pregnant underweight increased the incidence of fetal growth retardation [28]. The neonatal BL was lower in women with pregestational BMI<18.5 kg/m^2^ in comparison with women with pregestational BMI 18.5–25 kg/m^2^ [29]. GWG reflects nutritional status during pregnancy [30, 31]. Excessive GWG can increase the incidence of GDM, pregnancy complications, fetal distress, neonatal death, and macrosomia [32–36].

In our study, the BW, BL, CC, HC were significantly higher in male newborns than that in female newborns. Multivariate analyses in our study showed that the male newborns were more susceptible to macrosomia, long BL, large HC and CC. Male infants are more susceptible to the influence of the utero environment and absorb nutrients more efficiently and grow faster than female fetus [37]. Some studies have also found a gender correlation with neonatal BW was higher in male than female newborns [38, 39]. A study [40] in Nigeria showed that mean HC was higher in male neonates than that in females in all gestational age groups.

In our study, gestational age was observed to be a major factor affecting neonatal BW, BL, HC and CC in women with GDM. Therefore, it is very important for pregnant women with GDM to pay attention to the gestational age. ACOG recommends that fetal monitoring be considered starting at 32 weeks for pregnant women with GDM [8], unless other factors increasing fetal risk are present. About the timing and modality of delivery, ACOG suggests expectant management up to 40 + 6–7 weeks for women with diet only GDM and good glycemic control; instead, if GDM is well controlled by medications delivery is recommended between 39 and 40 weeks of gestation. Earlier delivery between 37+0 and 39 weeks is recommended for woman with poorly controlled GDM.

The study has some limitations. First, the sample size is relative small, the findings need to be replicated in further prospective cohort studies with larger samples. Second, dietary intakes were not included in the investigation, which might be a factor influencing the neonatal physical development.

## Conclusion

Our study found that gestational age, GDM status, neonatal gender, GWG, and pre-pregnancy BMI were associated with neonatal physical development.

GDM will increase the risk of macrosomia and large CC. Male neonate had a higher risk of macrosomia, long BL, large HC and large CC. Female neonate increased the risk of short BL and small HC. For women without GDM, pre-pregnant BMI and GWG are crucial for estimating the risk of neonatal abnormal physical developments. Our results suggest increasing pre-pregnancy BMI to the normal range can reduce the risk of short BL and small CC and reducing GWG to the normal range can reduce the risk of macrosomia, large CC and large HC. In women with GDM, gestational age was correlate with neonatal abnormal physical developments.

## Supporting information

S1 File (DOCX)

S2 File (DOCX)
