# Supplementary material for: The factors affecting the physical development of neonates in pregnant women with or without gestational diabetes mellitus
Source: PLoS One. 2021 Apr 30;16(4):e0251024. doi: 10.1371/journal.pone.0251024 (PMC8087091; doi:10.1371/journal.pone.0251024)
Supplement: S1 File — (DOCX) [file pone.0251024.s001.docx]

郑州大学第二附属医院孕妇及新生儿基本情况调查表

编号： ________

一、孕妇一般情况

1.1 年龄： _______岁

1.2 身高： ___cm

1.3 孕前体重：____kg

1.4 分娩前体重：____kg

1.5 民族： （1）汉族；（2）回族；（3）其他

1.6 居住地： （1）城市；（2）农村

1.7 文化程度： （1）初中及以下；（2）高中；（3）大专；（4）本科及以上

1.8 职业类别： （1）教育类；（2） 医疗卫生类；（3）商业类；（4）机关类

（5）其他（请注明） ________

1.9 您家庭的人均月收入为哪个水平？

（1）1000 及以下；（2）1001-2000；（3）2001-3000；（4）3001-4000；（5）4000 及以上

1.10 既往慢性病史（孕前）：（1）无；（2）心脑血管疾病；（3）肝脏肾脏疾病； （4）糖尿病；（5）其他（请注明） _________

1.11 家族病史：（1）无；（2）心脑血管疾病；（3）肝脏肾脏疾病；（4）糖尿病；（5）其他（请注明） _________

1.12 孕次： _____

1.13 产次： ____

1.14 分娩方式： 1.自然分娩 2.剖宫产

1.15 孕周： ____周

1.16 入院诊断：

二、生化检验

2.1 血压_____/______mmHg

2.2 脉搏______次/分

2.3血红蛋白水平： 1. 产前________g/L ；2.产后________g/L

2.4葡萄糖耐量试验：1.空腹血糖______mmol/L； 2.1 小时后血糖______mmol/L； 3. 2小时后血糖______mmol/L

2.5 糖化血红蛋白________%

2.6 尿素______mmol/L

2.7 肌酐_____mol/L

2.8 谷草转氨酶_______μ/L

2.9 谷丙转氨酶_______μ/L

三、新生儿的基本情况

3.1 性别： 1.男；2.女

3.2 体重_____g

3.3 身长______cm

3.4 胸围______cm

3.5 头围______cm

3.6 阿氏评分(Apgar)： 1分钟____分； 5分钟____分； 10分钟___分
