# Supplementary material for: The factors affecting the physical development of neonates in pregnant women with or without gestational diabetes mellitus
Source: PLoS One. 2021 Apr 30;16(4):e0251024. doi: 10.1371/journal.pone.0251024 (PMC8087091; doi:10.1371/journal.pone.0251024)
Supplement: S2 File — (DOCX) [file pone.0251024.s002.docx]

A survey on basic condition of pregnant women and newborns in 2^nd^ Affiliated Hospital of Zhengzhou University

Number:

1. **General situation of pregnant women**

1.1 Age: _______ years old

1.2 Height: ___cm

1.3 Pre-pregnancy body weight ____kg

1.4 Body weight at delivery ____kg

1.5 Nation (1) Ethnic Han; (2) ethnic Hui; (3) Other

1.6 Residential address (1) City; (2) Rural

1.7 Maternal education (1) Middle school/below; (2) High school; (3) Junior college; (4) Bachelor degree or above

1.8 Occupational (1) Education; (2) Medical and health; (3) Business; (4) Administrative; (5) Other

1.9 What is the per capita monthly income of your family (RMB) ?

(1) ≤ 1000 Yuan (2) 1001-2000 (3) 2001-3000 (4) 3001-4000 (5) ≥4000

1.10 History of chronic diseases (before pregnancy):

(1) No (2) Cardiovascular and cerebrovascular diseases (3) Liver and kidney diseases (4) Diabetes mellitus (5) Others

1.11 Family history of disease:

(1) No (2) Cardiovascular and cerebrovascular diseases (3) Liver and kidney diseases (4) Diabetes mellitus (5) other

1.12 How many times have you been pregnant? ____

1.13 How many times have you given birth? ____

1.14 Delivery pattern 1. Vaginal delivery 2. Cesarean ____

1.15 Gestational age _____ weeks

1.16 Hospital diagnosis:

二．**Biochemical test**

2.1 Blood Pressure_____ / ______mmHg

2.2 Pulse _____ times/min

2.3 Hemoglobin levels: 1. Ante partum ________g/L; 2. Post-delivery ________g/L

2.4 OGTT: 1. Fasting blood-glucose ______ mmol/L; 2. 1-hour plasma glucose following a 75 g oral glucose load ______ mmol/L; 3. 2-hour plasma glucose following a 75 g oral glucose load ______ mmol/L

2.5 Glycated hemoglobin _____ %

2.6 Urea _____ μmol/L

2.7 Creatinine _____μmol/L

2.8 AST (Aspartate aminotransferase) _____μ/L

2.9 ALT (Alanine transaminase) _______μ/L

三．**Basic information of newborns**

3.1 Gender: 1. Male 2. Female

3.2 BW_____ g

3.3 BL ______cm

3.4 CC ______cm

3.5 HC ______cm

3.6 Alzheimer's score (Apgar)

1 minute ____; 5 minutes ____; 10 minutes ___
